# Circular SERPINA3 and its target microRNA-944 as potential biomarkers in hepatitis C virus-induced hepatocellular carcinoma in Egyptian population

**DOI:** 10.1016/j.ncrna.2023.05.005

**Published:** 2023-05-23

**Authors:** Nora M. Aborehab, Mohamed A. Kandeil, Dina Sabry, Radwa Rabie, Ibrahim T. Ibrahim

**Affiliations:** a Department of Biochemistry, Faculty of Pharmacy, October University for Modern Sciences and Arts (MSA), Giza, 12451, Egypt; b Department of Biochemistry, Faculty of Veterinary Medicine, Beni-Suef University, Beni-Suef, 62521, Egypt; c Medical Biochemistry and Molecular Biology Department, Faculty of Medicine, Badr University in Cairo, Cairo, 11829, Egypt; d Medical Biochemistry and Molecular Biology Department, Faculty of Medicine, Cairo University, Cairo, 11562, Egypt; e Department of Biochemistry, Faculty of Pharmacy, Beni-Suef University, Beni-Suef, 62521, Egypt

**Keywords:** miR-944, Glypican-3, Metastasis, Hepatitis C, CircSERPINA3

## Abstract

**Background:**

The most prevalent cancer in Egypt is hepatocellular carcinoma (HCC) mainly due to the infection with the hepatitis C virus. So it is critical to find sensitive biomarkers for early diagnosis of HCC and avoid post-operation tumor recurrence. Therefore, this research was designed to demonstrate the circSERPINA3 role in the regulation of microRNA-944 gene expression in HCV-related HCC cases and compare these results with circSERPINA3 and microRNA-944 gene expression levels in HCV-infected patients.

**Methodology:**

Study participants were divided into three groups: healthy controls, HCV- infected, and HCV-induced HCC patients. The gene expression levels of circSERPINA3 and microRNA-944 were evaluated using Real-Time qPCR. Then the immunoblotting procedure was applied to measure the serum levels of MDM2 and E-cadherin besides, the serum concentration levels of glypican-3 and alpha-fetoprotein were measured by sandwich ELISA.

**Results:**

The gene expression level of circSERPINA3 was significantly upregulated in both HCV-infected and HCC patients causing suppression of the antitumor effect of miR-944 and showing a lower 1-year survival rate than the participants who had low circSERPINA3 gene expression levels. Subsequently, the miR-944 downstream protein, MDM2 was remarkably upregulated, exaggerating the metastasis and oxidative stress in HCC cases. Additionally, the results confirmed the downregulation of microRNA-944 improved the progression of viral hepatitis C cases to hepatocarcinogenesis through the significantly increased serum level of the metastatic marker, E-cadherin. Although alpha-fetoprotein is a common diagnostic marker used in the diagnosis of HCC, our results showed that glypican-3 had greater sensitivity and specificity and positively correlated to the IGF-1 signaling pathway of HCC cases. Moreover, the gene expression levels of circSERPINA3 and E-cadherin in both the HCV and HCV-induced HCC were significantly positively correlated.

**Conclusion:**

circSERPINA3 and miR-944 were sensitive molecular markers for early diagnosis of HCC and could be prospective treatment targets for HCV-infected patients to avoid tumor recurrence in HCC cases.

## Highlights

• HCC patients are in front of early tumor recurrence after tumor resection.
• CircSERPINA3 enhanced the HCV progression to HCC by miR-944 sponging.
• MDM2 is a downstream gene of miR-944 that targets IGF-1R/Akt and inhibits the HCC.
• Inhibition of miR-944 exaggerates the risk of HCC metastasis through increased E-cadherin.
• GPC3 is more sensitive and accurate than AFP in HCC diagnosis.

## Graphical abstract

**Figure:**
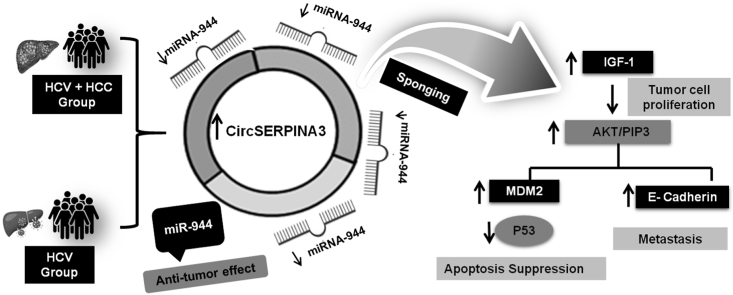

## 1 Introduction

Hepatocellular carcinoma (HCC) is the third principal reason of death worldwide; in addition, it ranks as the fourth most frequent cancer in Egypt. The risk of developing HCC is raised by twenty folds in presence of the chronic hepatitis C infection because 40%–50% of HCC cases in Egypt are primarily caused by the infection with hepatitis C [1,2].

There is a direct correlation between chronic HCV infection and the development of HCC, suggesting that HCV viral infection is oncogenic. When the HCV replication cycle occurred, certain viral proteins and replication intermediates were identified as pathogen-associated molecular patterns (PAMPs) that activate the innate and adaptive immune systems [3] and promote the E-cadherin liberation from hepatocytes. All these events stimulate the release of anti-apoptotic signals and the progression of HCC metastasis. So, HCV- viral infection is one of the major causes of HCC with high mortality and morbidity rates [4].

While sorafenib and regorafenib medications are regarded as the first and second treatment lines of HCC in advanced cases, respectively, as approved by the US FDA, tumor control therapies, surgical resection, liver transplantation, and radiotherapies are effective in the treatment of early stages of HCC. Nonetheless, 80% to 90% of individuals who have had hepatocellular carcinoma treated are at significant risk of experiencing a five-year HCC recurrence, adverse effects from the current therapies, and drug resistance [5].

Early diagnosis of HCC by more specific accurate biomarkers and investigation of the molecular mechanisms underlying the HCC pathogenesis and progression are very important in discovering the therapeutic targets of this cancer type [6]. Non-coding RNAs, the major components of the human transcriptome, are considered the promising treatment and diagnostic tools of HCC cases with high tumor recurrence survival [7]. Non-coding RNAs are the untranslated parts of the genome sequence and thousands of them have important functions in cell homeostasis like epigenetic and translational modifications. For example, PARS, siRNA, and piRNAs play important roles in gene expression regulation and miRNAs regulate the gene post-transcriptional events while siRNAs and lncRNAs are involved in alternative splicing and translational regulation [8]. According to previous studies, the accumulation of RNA/DNA hybrids at diverse promoters, enhancers, and genes might result in somatic DNA mutagenesis that may be carcinogenic. Rapid RNA degradation, which is controlled by MTR4 and the RNA exosome complex, prevents these changes. The ncRNAs may be marked for survival or destruction depending on whether they associate with RNA-binding proteins such as splicing factors or creating secondary RNA structures. For example, nuclear m6A-modified RNA reader YTHDC1 and the RNA exosome cofactor MPP6 cooperate together to attract the RNA exosome complex to the IgH switch region germline transcript of lncRNA (SGμLT) to facilitate processing and programmed DNA recombination in B cells [9]. Several miRNAs are dysregulated in hepatocellular carcinogenesis and targeting these miRNAs in the treatment is considered a promising therapeutic strategy. MicroRNAs play dual roles in malignancy, as tumor suppressors or promoters. For instance, miRNAs- 645, 34a, and 506 are downregulated in HCC while miRNAs- 650, 552, and 197 are upregulated [10].

The miRNA-944 role in malignancy is tissue specific; it is downregulated in gastric and colorectal cancers indicating poor prognosis and highly expressed in breast and cervical cancers [[11], [12], [13], [14]]. MicroRNA-944 is a valuable biomarker for the prediction of hepatocellular carcinoma.

miR-944 acts on IGF-1R/ PI3K/Akt pathway. IGF-1R is activated by IGF-1 which induces the phosphorylation of the kinase receptor. This oncogenic pathway (IGF) stimulates tumor cell proliferation and inhibits apoptosis. Also, the activation of the IGF pathway upregulates PI3K, RAF/MEK/ERK, and protein kinase-B hepatocellular carcinoma signaling pathways. In the cytoplasm, phosphoinositide 3-kinase (PI3K) generates phosphoinositol triphosphatewhich (PIP3b) which upregulates the AKT. Also, AKT kinase could be activated by phosphorylation with GPC-3 and FGF-2 which promotes the cell cycle progress from G1 to S phase [15].

As the majority of solid tumors developed from epithelial tissue, E-cadherin plays a crucial role in epithelial cell adhesion and the loss of its function is a significant contributor to metastasis. Overexpression of miR-944 stimulates apoptosis and suppresses tumor cell proliferation and metastasis in HCC patients by suppressing of E-cadherin levels and inhibiting the insulin growth factor-1 receptor (IGF-1R) gene expression that stimulates the phosphatidylinositol-3 kinase and protein kinase B (PI3K/Akt) leading to very poor survival of HCC patients [16]. So, suppression of miR-944 gene expression exaggerates the metastasis through increased E-cadherin levels causing poor prognosis. E-cadherin plays a vital role in the progression of HCV infection into HCC. The cell-surface distribution of CLDN1 and OCLN, two important tight junction proteins, as well as HCV co-receptors, is regulated by E-cadherin expression. This disturbance increases the cell motility and exaggerates the cadherin-catenin complex formation causing increased potential to develop metastasis [17].

In most cases, overexpression of circRNAs inhibits the actions of microRNAs to decrease the translation of mRNAs that encode oncogenic proteins by sponging and interference in the cellular signaling pathways that play an important role in controlling cell proliferation, metastasis, and apoptosis. Circular RNA has a distinctive role in the regulation of tumor cell proliferation, metastasis, and apoptosis, such as in gastric cancer; it promotes tumor cell proliferation [18]; in contrast, circMTO-1 reduces cell proliferation in colorectal cancer [19].

However, there are limited research studies on circSERPINA3 role in different types of malignancies. Previous studies showed that circSERPINA3 has a microRNA-944 binding sequence and could suppress the action of microRNA-944 by sponging. CircSERPINA3 upregulates the miR-944 downstream protein (MDM2) expression levels, inhibiting the anti-metastasis effect of P53 and exaggerating oxidative stress. This action is reversed by microRNA-944 which is involved in hepatocellular carcinoma therapy [20].

IGF-1R overexpression is commonly seen in tumors and is a sign of poor prognosis in people with HCC. IGF/IGF-1R signaling promotes tumor cell invasion and metastasis by regulating cell growth, survival, migration, and protein synthesis. It can also prevent apoptosis by expressing Myc and Akt1 in the liver. IGF-1R expression can be inhibited by the tumor suppressor p53 by blocking IGF-1R transcription [21].

Glypican-3 is only greatly expressed in non-cirrhotic hepatocellular carcinoma cases. It has an oncogenic effect by promoting the IGF-1R action through the same pathway of microRNA-944. The high levels of miR-944 gene expression deactivate the oncogenic pathway of IGF-1R; in contrast, glypican-3 upregulation has an oncogenic effect and decreases the IGF-1R ubiquitination and degradation [22].

Therefore, this research was designed to demonstrate the circSERPINA3 role in the regulation of microRNA-944 gene expression in HCC cases, and compare the results with microRNA-944 gene expression in HCV-infected patients. For this purpose, the gene expression levels of circSERPINA3 and miR-944 were measured in addition to, the blood concentration of MDM2 was measured to assess the liver oxidative stress and metastasis status and the concentration of E-cad was measured to evaluate the cell proliferation and tumor metastasis. Additionally, the plasma concentration levels of AFP and GPC3 were measured to determine the diagnostic accuracy of GPC3 in HCC patients.

## 2 Subjects and methods

### 2.1 Study population

The design of the research was a retrospective study, including 124 participants; 70 adult Egyptian patients with hepatocellular carcinoma, 28 adult Egyptian patients with viral hepatitis C infection, and 26 adult Egyptian control subjects as shown in (Table 2) at the Multidisciplinary clinic, Cairo University, Kasr Al- Ainy Hospital. The enrolled participants’ age was more than 18 years old and both sexes were included in the study. All hepatitis C virus and HCC cases were fitted for interventional standard therapies and positive for hepatitis C RNA. HCC patients were diagnosed by (CT Scan) or (MRI Scan). In addition, all the patients showed negative results to the Hepatitis B qualitative serologic tests (HBsAg, HBsAb & HBVDNA). However, patients who had ascites; other types of liver cancer, HBV, or any other detectable cause of chronic hepatitis besides HCV infection and severe psychiatric disease were excluded from the research. Also, all the enrolled patients who were cannabis addicted, smokers or alcoholics, or exposed to aflatoxin and heavy metals toxicity were excluded from this study. Moreover, Fibroscan (non-invasive method) was used to exclude patients suffering from NAFL & NASH. This research was agreed upon by the local ethical board in Kasr Al-Ainy Hospitals according to the ethical guiding principles of the 1975 Declaration of Helsinki. All participants signed the informed consent prior to the donation of blood according to the ethical standards of the Helsinki Declaration and current national laws, as well as approved by the Research Ethics Committee at the Faculty of Pharmacy, MSA University, Cairo, Egypt. (Approval number: Bp1/EC1/2020MSC).

### 2.2 Blood sample collection

Five milliliters of venous blood samples were collected from antecubital vein under sterile conditions for all the study participants for routine blood tests besides, 3 ml were taken for the assessment of miR-944 and circSERPINA3 gene expression levels and the rest of the plasma was collected for MDM2, E-cadherin, glypican-3 and AFP protein levels.

Total RNA was extracted from peripheral mononuclear cells (PMNCs) of subjects’ peripheral plasma in all different groups by double centrifugation (3500xg for 15 minutes at 5^o^C followed by centrifugation by 15000xg rate for 5 minutes at 5^o^C). Therefore, ncRNAs are protected from RNA exosome complex degradation [9].

(600 μl) of RNA lysis buffer was added to (200 μl) of collected mononuclear cells of blood and stored at −80 °C till assessment of RNA extraction. Then, the rest of the whole blood samples were centrifuged at 4000 rpm for 10 min and (400 μl) of radioimmunoprecipitation assay (RIPA) buffer was added to (200 μl) of separated plasma and stored at −80 °C till assessment of MDM2 and E-cadherin protein expression levels. Finally, the reminder plasma was collected and separated for alpha-fetoprotein (AFP) and glypican-3 (GPC3) assessment by ELISA.

### 2.3 Methods

#### 2.3.1 Quantitative mRNA expression of circSERPINA3 and miRNA944: were analyzed by real-time PCR (qRT-PCR) technique

RNA Miniprep Plus (ZYMO RESEARCH CORP, USA) was applied to separate the RNA from peripheral mononuclear cells (PMNCs) from the patients’ peripheral blood of the three groups and the samples quality and amount were evaluated by Beckman dual spectrophotometer (USA). By one step qRT-PCR, the cDNA was synthesized from the RNA extracted from every sample with a Reverse Transcriptase kit (Applied Biosystem, USA). The free ncRNAs are exposed to RNA exosome complex degradation and could be affected by gene expression modifications leading to changes in their cellular proliferation functions (Nair et al., 2021). Therefore, All RNA samples were extracted from mononuclear cells (MNCs) from peripheral blood to be protected against the RNA exosome complex. By one step qRT-PCR, the cDNA for miR-944 and the cDNA for circSERPINA3 were synthesized from the total RNA extracted from every sample with a Reverse Transcriptase kit (Applied Biosystem, USA). Then, the cDNA for miR-944 and cDNA for circSERPINA3 samples size were amplified by using the Syber Green I PCR Kit (Applied Biosystem, USA). After the reverse transcription, a quantitative PCR reaction was performed using SYBR Green dye emits fluorescence when bound to double-stranded DNA [9]. The sequences of the primers are mentioned in (Table 1).

**Table 1:** Primers sequence of all studied genes.

	Forward sequence	Reverse sequence
*miR-944*	GCGGCGGAAATTATTGTACATC	ATCCAGTGCAGGGTCCGAGG
*circSERPINA3*	TGCAGAAAGGAGGGTGATTT	GGCCTCCTGACAGCAATAAA
*U6*	ACGAATTTGCGTGTCATCCTTGCG	TCTCGCTTCGGCAGCACAT ATACTA
*β-actin*	GGCGGCACCACCATGTACCCT	AGGGGCCGGACTCGTCATACT

The RQ of each target gene is quantified and normalized to the housekeeping gene according to the calculation of delta-delta Ct (ΔΔCt). We calculated the RQ of each gene by taking 2^-ΔΔCt^ as follows: ΔΔCt = [(Ct target, Sample)-(Ct reference, Sample)]-[(Ct target, Control)-(Ct reference, Control)].

#### 2.3.2 Quantitative immunoblotting assessment of MDM2 and E-cadherin

The proteins were separated from each sample by Ready Prep TM protein extraction kit (Bio Rad Inc, USA), by RIPA lysis buffer (Bio BASIC INC, Canada) with additional protease inhibitor and phosphatase inhibitor buffer. Then, the samples were centrifuged at ∼16,000×g for 30 minutes at 4°C to separate the supernatant and the amount of the sample was measured with the Bradford Assay Kit (Bio Basic Inc, Canada). After the separation of the antigen samples by sodium dodecyl sulfate-polyacrylamide, gel electrophoresis (SDS-PAGE) gel (Bio-Rad Inc, USA) to the immunoblotting membrane (PVDF) membrane blocked with 3% bovine serum albumin (BSA) using Bio-Rad Trans-Blot Turbo. The MDM2 and E-cadherin proteins were incubated with their diluted primary at 4°C overnight and washed with TBST and secondary antibodies conjugated with HRP enzymes. Then, chemiluminescent signals were measured and the bands were analyzed using ChemiDoc MP Imager against beta-actin [23].

#### 2.3.3 Estimation of glypican-3 & alpha-fetoprotein concentration levels

GPC3 and AFP concentrations were estimated by the ELISA technique provided by (Cat# Number DGLY30) Quantikine® ELISA (USA & Canada) and (Cat# Number DAFP00) Quantikine® ELISA (USA & Canada), respectively. The 96-well microplates were pre-coated with monoclonal antibodies specific to GPC3 and AFP proteins overnight at 4°C. Then, 100 μL of assay diluent (buffered protein base) was added to 100 μL of standard, control, or sample in each well to be incubated for two hours on an orbital microplate shaker, and the wells were aspirated and washed four times with the wash buffer. After the last wash step, the entire wash buffer was removed by aspiration and the microplate wells were inverted over a clean towel. Afterward, the plates were blocked for two hours with 1% BSA. After that, 200 μL of the polyclonal antibodies specific to the protein samples conjugated to HRP enzymes were added; every well was covered with an adhesive strip and incubated for two hours at room temperature. Subsequently, the washing step was repeated again then 200 μL of substrate solution was added to each well and incubated for half an hour away from the light, and color developed in proportion to the amount of the protein sample concentration. Finally, 50 μL of stop solution (2 N sulfuric acid) was added to all wells, tapped for mixing and the wells' colors changed from blue to yellow. The absorbance of each well was determined within 30 minutes at 450 nm, using an ELISA microplate reader [24].

### 2.4 Statistical analysis

The difference between the three studied groups was evaluated using one-way ANOVA and Tukey Kramer's multiple comparisons tests. The diagnostic accuracy of tested markers was assessed by ROC analysis. Pearson correlation was applied to evaluate the correlation capacity between the circSERPINA gene expression and the different measured markers in patients' plasma. χ2 (chi-squared) test was used to compare between groups for categorical variables, as appropriate. Kaplan–Meier analysis was used to determine the overall survival (OS). A two-tailed test with a P-value less than 0.05 was considered statistically significant. The statistics of this research were done using GraphPad Prism 6 (GraphPad Software, California, USA).

## 3 Results

### 3.1 Clinical characteristics of patients and controls

124 participants were enrolled in the research. 70 patients with hepatocellular carcinoma, 28 patients with viral hepatitis C infection, and 26 control subjects with their ages ranging from 59 to 61 years. The clinical characteristics of the research enrolled members were described as shown in (Table 2).

**Table 2:** Characteristics of patients and controls. Data are expressed as mean ± SEM or n (%). The clinical data were analyzed by ANOVA and Tukey Kramer's multiple comparison tests. Gender was analyzed by X^2^ test.^a^ statistically significant from healthy controls.^b^ statistically significant from HCV. WBCs, white blood cells; ALT, alanine aminotransferase; AST, aspartate aminotransferase; ALP, alkaline phosphatase; T Bil, total bilirubin; AFP, alpha-fetoprotein.

Variables	Control (n = 26)	HCV (n = 28)	HCV-induced HCC (n = 70)	*P-* value
Age (Years)	59.65 ± 0.8520	60.14 ± 0.8062	61.24 ± 0.6124	
Gender (Male/Female)	14 (53.84%) 12 (46.15%)	13 (42.8%) 15 (57.14%)	37 (52.85%) 33 (47.14%)	
Hemoglobin (g/dL)	13.97 ± 0.1874	12.74 ± 0.2113^a^	11.37 ± 20.1968^ab^	0.0008
WBCs ( × 10^3^/mm^3^)	5.875 ± 0.2707	5.711 ± 0.2048	5.601 ± 0.2140	0.5228
Platelets ( × 10^3^/mm^3^)	273.2 ± 17.14	223.9 ± 11.92^a^	132.8 ± 7.640^ab^	<0.0001
ALT (IU/L)	27.31 ± 1.317	55.10 ± 3.626^a^	61.50 ± 5.793^a^	<0.0001
AST (IU/L)	25.50 ± 1.200	55.57 ± 4.826^a^	68.78 ± 4.289^a^	<0.0001
ALP (IU/L)	56.88 ± 4.076	109.4 ± 12.53^a^	161.8 ± 17.16^ab^	<0.0001
Total Bil. (mg/dl)	0.5318 ± 0.04167	0.9636 ± 0.05104^a^	1.680 ± 0.1257 ^ab^	<0.0001
Albumin (g/dl)	4.357 ± 0.08347	3.828 ± 0.1154^a^	3.331 ± 0.07389^ab^	<0.0001
Creatinine (mg/dl)	0.8692 ± 0.06511	0.9254 ± 0.03792	1.104 ± 0.1690	0.6977
AFP (ng/ml)	4.639 ± 0.5528	12.83 ± 5.589^a^	8621 ± 3621^ab^	<0.0001

### 3.2 Comparison and correlation of the gene expression levels of circSERPINA3 and miR-944 among the studied groups

The upregulation of circSERPINA3 level and its effect on the downregulation of miR-944 gene expression level in HCV and HCV-induced HCC groups’ plasma indicated a poor prognosis.

**Fig. 1:**
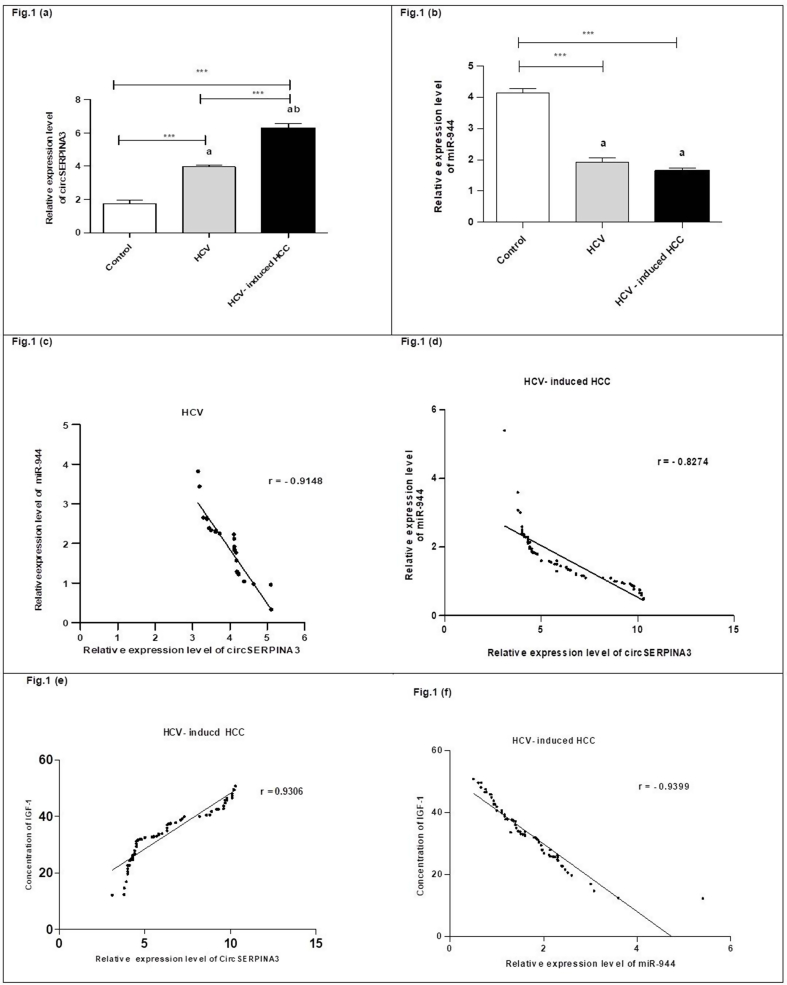
Relative gene expression levels in different studied groups. (a, b) Gene expression levels of circSERPINA3 and miR-944 in the three studied groups' plasma were measured by RT-qPCR. **(c)** The negative correlation between circSERPINA3 expression and miR-944 expression in the HCV group. **(d)** The negative correlation between miR-944 expression and circSERPINA3 expression in HCV-induced HCC group. **(e)** The positive correlation between circSERPINA3 and IGF-1 in HCV-induced HCC group. **(f)** The negative correlation between miR-944 and IGF-1 in HCV-induced HCC group. Gene expression levels are expressed as mean ± SE. The data were analyzed using ANOVA and Tukey Kramer's multiple comparison tests. a statistically significant from healthy controls. b statistically significant from HCV. ***Indicates significance at p < 0.001. HCV, hepatitis C virus; HCC, hepatocellular carcinoma.

Gene expression level of circSERPINA3 was remarkably higher in HCV and HCC groups in comparison to the study controls. Moreover, circSERPINA3 gene expression level was significantly higher in the HCV-induced HCC group than in the HCV group as shown in (Table S1) (see Fig. 1a). In addition to the gene expression level of miR-944 was significantly suppressed in HCV and HCV-induced HCC groups compared to the corresponding controls, but didn't reach the statistically significant difference between the HCV and HCV-induced HCC groups as shown in (Table S1) (see Fig. 1b).

There were significant negative correlations between the gene expression levels of circSERPINA3 and miR-944 in the HCV group (r = −0.9148) (see Fig. 1c) and in the HCV-induced HCC group (r = −0.8274) (see Fig. 1d). Besides, IGF-1 level was remarkably positively correlated to the circSERPINA gene expression level (r = 0.9306) (see Fig. 1e) and significantly negatively correlated to miR-944 gene expression levels (r = −0.9399) (see Fig. 1f).

### 3.3 Survival rate for HCC patients in relation to circSERPINA3 gene expression

**Fig. 2:**
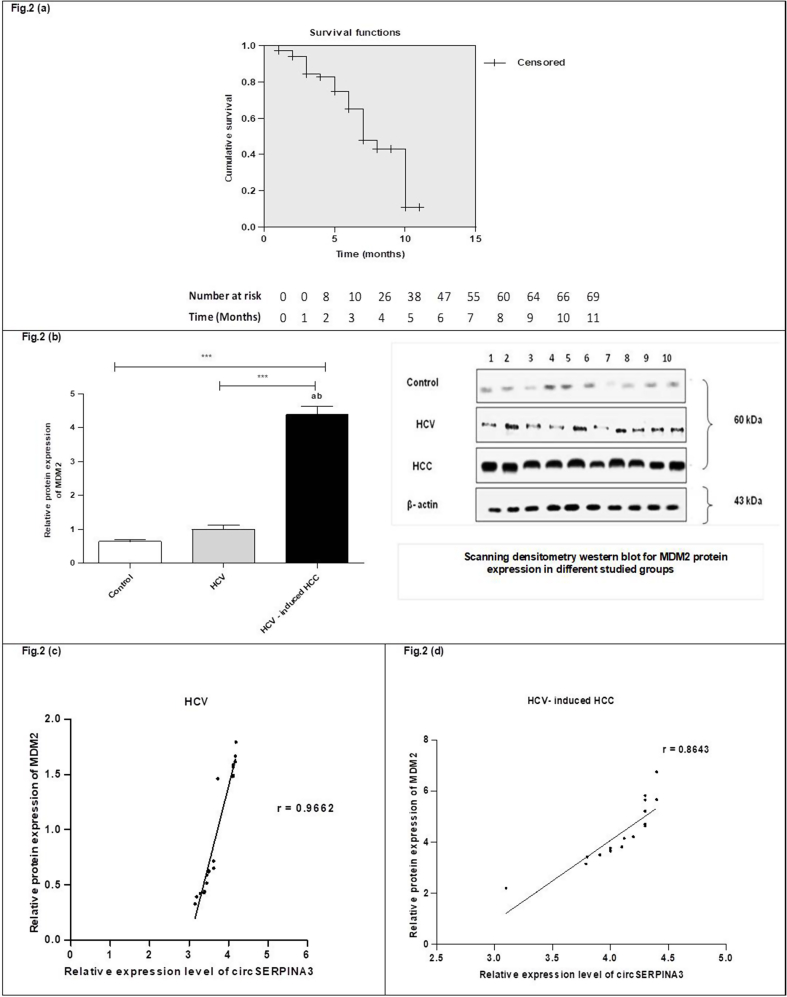
Plasma relative MDM2 protein expression levels in different studied groups. **(a)** Kaplan-Meier survival curve for HCC patients in relation to circSERPINA3 gene expression. **(b)** The protein expression level of MDM2 in the three studied groups' plasma was detected by western blotting analysis. **(c)** The positive correlation between circSERPINA3 expression and MDM2 expression in HCV group. **(d)** The positive correlation between circSERPINA3 expression and MDM2 expression in HCV-induced HCC group. Protein expression levels are expressed as mean ± SE. The data were analyzed using ANOVA and Tukey Kramer's multiple comparison tests for comparing the three studied groups. a statistically significant from healthy controls. b statistically significant from HCV. ***Indicates significance at p < 0.001. HCV, hepatitis C virus; HCC, hepatocellular carcinoma; MDM2, mouse double minute 2 homolog.

HCC patients with a significant increase in circSERPINA3 gene expression showed a considerable lower 1-year survival rate, about 10.75% of HCC patients survived (as shown in Fig. 2a).

### 3.4 Comparison of MDM2, E-cadherin, and Glypican-3 proteins levels and the correlation between the gene expression level of circSERPINA3 and the proteins levels among the studied groups

MDM2 protein expression level was greatly raised in the HCV-induced HCC group in comparison to the HCV group and the corresponding controls but, didn't reach a statistical significance difference between the HCV cases and the controls as shown in (Table S2) (see Fig. 2b). Moreover, there were significant positive correlation between the gene expression level of circSERPINA3 and the MDM2 protein level in both the HCV (r = 0.9662) (see Fig. 2c) and the HCC cases (r = 0.8843) (see Fig. 2d) groups.

**Fig. 3:**
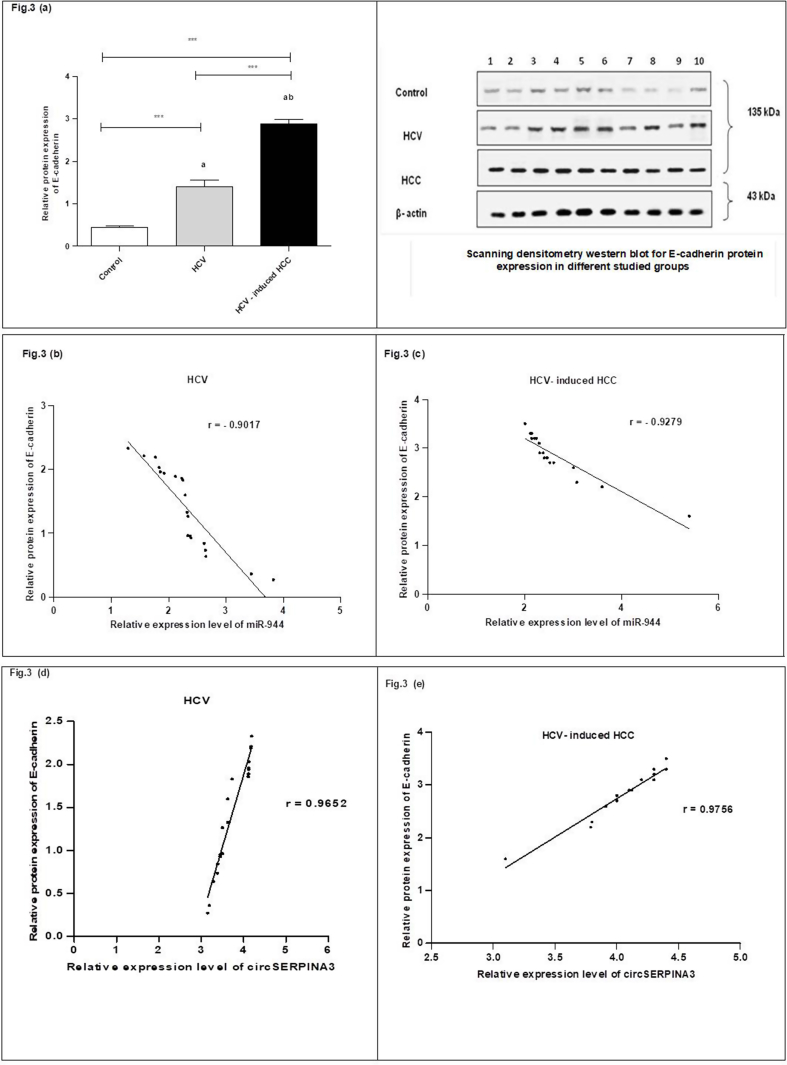
Relative protein expression level of E-cadherin among the studied groups. (a**)** The protein expression level of E-cadherin in the three studied groups' plasma was detected by western blotting analysis. **(b)** The negative correlation between miR-944 expression and E-cadherin expression in the HCV group. **(c)** The negative correlation between miR-944 expression and E-cadherin expression in HCV-induced HCC group. **(d)** The positive correlation between circSERPINA3 expression and E-cadherin expression in the HCV group. **(e)** The positive correlation between circSERPINA3 expression and E-cadherin expression in HCV-induced HCC group. Protein expression levels are expressed as mean ± SEM. The data were analyzed using ANOVA and Tukey Kramer's multiple comparison tests for comparing the three studied groups. a statistically significant from healthy controls. b statistically significant from HCV. ***Indicates significance at p < 0.001. HCV, hepatitis C virus; HCC, hepatocellular carcinoma.

Western immunoblotting demonstrated that the concentration level of plasma E-cadherin was considerably elevated in the HCC group as opposed to the HCV group and the corresponding controls, as well as the expression level of the protein in HCV patients, was significantly higher than the corresponding controls as described in (Table S2) (see Fig. 3a). Also, there were significant negative correlations between the gene expression levels of miR-944 and the plasma levels of E-cadherin in the HCV (r = −0.9017) (see Fig. 3b) and the HCC cases (r = −0.9297) (see Fig. 3c). In addition, the positive correlation between the gene expression level of circSERPINA3 and the E-cadherin protein level in both the HCV (r = 0.9652) (see Fig. 3d) and HCV-induced HCC (r = 0.9756) (see Fig. 3e) groups indicated that HCV-infected patients are at high risk of developing HCC and the HCC patients had poor prognoses with more chances for metastasis.

**Fig. 4:**
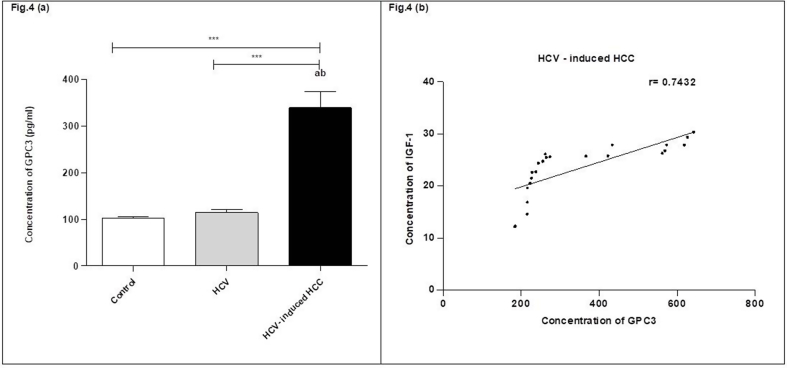
glypican-3 concentration among the studied groups. (a) The concentration of GPC3 in the three studied groups' plasma was detected by ELISA. **(b)** The positive correlation between the concentrations of GPC3 and IGF-1 in HCV- induced HCC group. Protein levels are expressed as mean ± SEM. The data were analyzed using ANOVA and Tukey Kramer's multiple comparison tests for comparing the three studied groups. a statistically significant from healthy controls. b statistically significant from HCV. ***Indicates significance at p < 0.001. HCV, hepatitis C virus; HCC, hepatocellular carcinoma; GPC3, glypican3; IGF-1, insulin-like growth factor 1.

The glypican-3 gene expression level, measured by sandwich ELISA, was obviously elevated in the HCC group when compared to the HCV group and the corresponding controls. However, there was no considerable difference in the expression level of GPC3 in the HCV group in comparison to the corresponding controls as clarified in (Table S2) (see Fig. 4a). The glypican-3 protein level was positively correlated to the plasma IGF-1 level (r = 0.7432) in HCC cases (see Fig. 4b).

### 3.5 Diagnostic performance of circulating circSERPINA-3, miR-944 and E-cadherin in HCV group

**Fig. 5:**
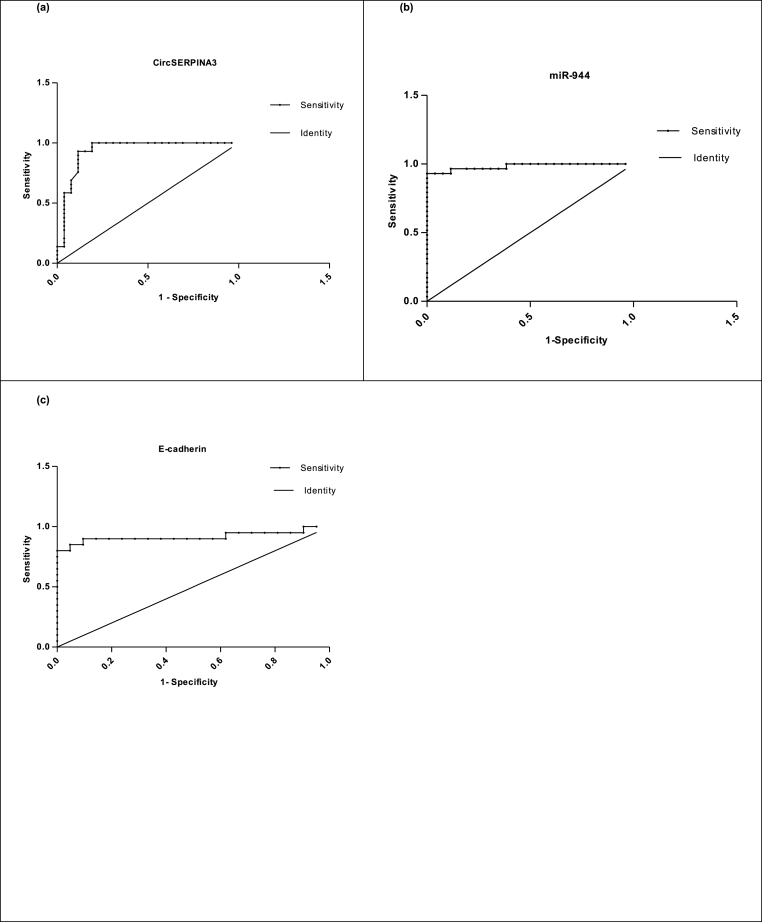
Receiver-operator characteristic (ROC) curves of the (a) CircSERPINA3, (b) miR-944, (c) E-cadherin, for the HCV group. HCV, hepatitis C virus; HCC, hepatocellular carcinoma.

The ROC curve analysis was used to analyze the diagnostic accuracy of the investigated plasma circSERPINA3, miR-944, and E-cadherin for the HCV group; the AUC values (Table 3) were analyzed to calculate the sensitivity and specificity (Table 3) (See Fig. 5a, b, c) respectively. ROC curve analyses revealed that the previous circulating markers could serve as valuable biomarkers for chronic hepatitis C from healthy controls with high significant sensitivity and specificity.

**Table 3:** The area under the Curve (AUC), Confidence Interval (CI), p-values, sensitivity, and specificity for the circulating diagnostic markers of the HCV group.

Variable(s)	AUC	95% Confidence Interval	Cut-off value	Sensitivity (%)	Specificity (%)
**CircSERPINA3 level** (HCV vs. Control)	0.935 ± 0.0379	0.8607 to 1.009	>2.825	100	80.77
**miR-944 level** (HCV vs. Control)	0.982 ± 0.0147	0.9538 to 1.012	<2.900	93.1	100
**E-cadherin level** (HCV vs. Control)	0.9167 ± 0.05319	0.8124 to 1.021	>0.6280	90.00	90.48

### 3.6 Diagnostic performance of circulating circSERPINA-3, miR-944, GPC3 and AFP in HCV induced HCC group

**Fig. 6:**
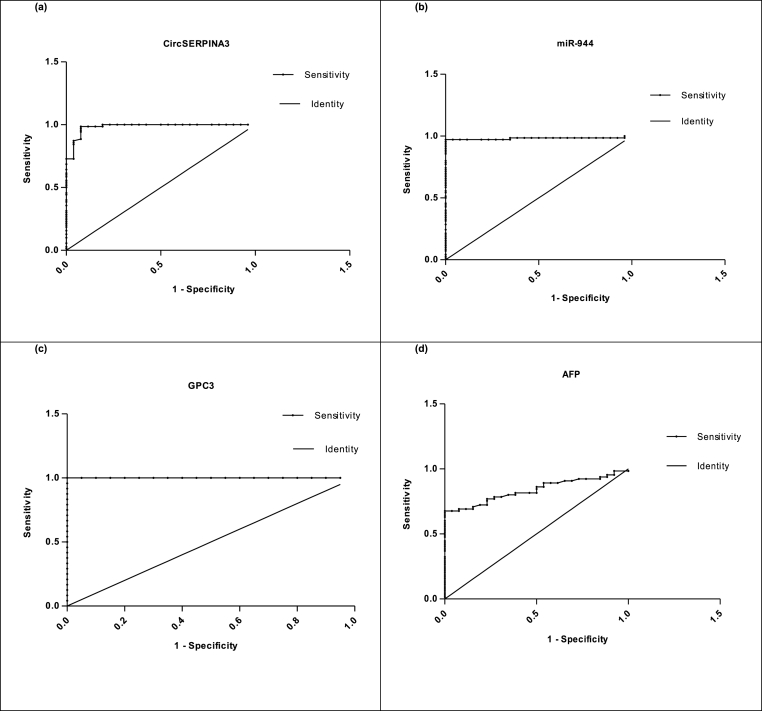
Receiver-operator characteristic (ROC) curves of the (a) CircSERPINA3, (b) miR-944, (c) GPC3, (d) AFP for the HCV-induced HCC group. HCV, hepatitis C virus; HCC, hepatocellular carcinoma; GPC3, glypican3; AFP, alpha-fetoprotein.

The ROC curve analysis was used to analyze the diagnostic accuracy of the investigated plasma circSERPINA3, miR-944, GPC3, and AFP for HCV-induced HCC group, the AUC values (Table 4) were analyzed to calculate the sensitivity and specificity (Table 4) (See Fig. 6a, 6b, c, d) respectively. ROC curve analyses revealed that the previous circulating markers could serve as valuable biomarkers for HCV-induced HCC from healthy controls. GPC3, circSERPINA-3, and miR-944 showed higher AUC, as well as higher sensitivity and specificity than the AFP in HCC cases.

**Table 4:** Area under the Curve (AUC), Confidence Interval (CI), p-values, sensitivity and specificity for the circulating diagnostic markers HCV induced HCC group.

Variable(s)	AUC	95% Confidence Interval	Cut-off value	Sensitivity (%)	Specificity (%)
**CircSERPINA3 level** (HCC vs. Control)	0.982 ± 0.012	0.9588 to 1.007	>2.800	100	80.77
**miR-944 level** (HCC vs. Control)	0.981 ± 0.014	0.9527 to 1.010	<3.115	97.14	100
**GPC3 level** (HCC vs. Control)	1	1.000 to 1.000	>157.4	100	100
**AFP level** (HCC vs. Control)	0.837 ± 0.0404	0.7580 to 0.9166	>10.15	67.69	96.15

## 4 Discussion

Hepatocellular carcinoma is highly prevalent worldwide due to chronic viral infections with a 5-year overall survival (>15%) particularly when diagnosed at the late stage of the tumor progression. According to Hassan et al. (2023), the non-invasive CT and MRI scans depend mainly on the tumor size for HCC diagnosis with very low sensitivity to the small sizes nodules, in addition to the standard therapies of HCC had many side effects. So, early diagnosis of hepatocellular carcinoma (HCC) by more specific accurate molecular biomarkers is critical for HCC treatment [25].

Current investigations approved that circular RNAs play many valuable roles in different biological processes whether normal or pathological, like hepatocarcinogenesis. As stated by Li et al. (2019), the main mechanism of circular RNA is sponging which could have oncogenic or tumor suppressor effects [26]. The hepatocellular carcinoma pathogenesis and the progression of this tumor type to metastasis could be related to the dysregulation of microRNA functions that developed by the increase or decrease of circular RNA gene expressions. So, studying the molecular pathways underlying hepatocarcinogenesis shows a crucial role in the early detection of the tumor where, these molecular markers could be valuable curative and early diagnostic targets for HCC [27]. For example, circLARP4 is highly expressed in HCC tissues leading to inhibition of p53 and p21 actions resulting from miR-761 sponging [28], He et al. (2020) approved that the increased concentrations of circ_0000517 in HCC tissues caused increased levels of IGF-1R due to sponging of miR-326 and enhancing the hepatocarcinogenesis [29].

This study aimed to investigate the circSERPINA3 role in the regulation of microRNA-944 gene expression in HCC cases and compare the results with microRNA-944 gene expression in HCV-infected patients.

Various circular and long non-coding RNAs compete with miR-944's targeted suppression action of mRNA to regulate the characteristics of cancer cells, including the cancer cell cycle, growth, proliferation, epithelial-mesenchymal transition (EMT), cancer cell invasion, and metastasis. The key signaling pathway that miR-944 targets and inhibits protein-coding genes is PI3K/AKT pathway [30]. The abnormal gene expression of miRNAs has been proposed as therapeutic targets for HCC patients as well as potential indications for diagnosis and prognosis, However, the studies examining the specific functions of miR-944 in HCC and their expression patterns are relatively limited. Because of this, it is vital and crucial to have a strong understanding of the fundamental mechanisms through which miRNAs impact HCC [30]. As a result, we examined the expression of miR-944 in HCC as well as looked at the molecular pathways behind miR-944's tumor suppressor effect in HCC and its relation to the gene expression of other non-coding RNAs. MicroRNA-944 has an anti-oncogenic effect through stimulation of apoptosis and decreases tumor cell proliferation, metastasis, and clone formation in HCC patients by inhibiting the insulin growth factor -1R gene expression [31].

According to Lv et al. (2019), upregulation of miR- 944 gene expression level has a malignancy suppression effect on HCC cells through suppression of tumor cell proliferation besides, augmentation of apoptosis by targeting the oncogenic tyrosine kinase receptor IGF-1R and deactivation of PI3K/Akt signaling mechanisms [16]. Ngo et al. (2022), found that IGF-1R contributes to the insistent malignant features and poor prognosis of HCC, in addition to chemotherapy resistance that could be reversed by downregulation of its gene expression through binding of microRNA-944 to 3′ prime end of IGF-1R to inhibit the gene translation [21].

The cellular biological functions of hepatocytes are closely related to the serpins family which includes SERPINA3, and it is extensively expressed in human organs [32]. Liu et al. (2020) study confirmed that circSERPINA3 gene expression level was upregulated in nasopharyngeal carcinoma aggravating the cancer progression effects of MDM2 (downstream gene of microRNA-944), cell invasion and proliferation, through sponging of the tumor suppressor microRNA-944 [20].

In most cases, overexpression of circRNAs inhibits the actions of microRNAs to decrease the translation of mRNAs that encode oncogenic proteins by sponging and interference in the cellular signaling pathways that play an important role in controlling cell proliferation, metastasis, and apoptosis [20].

The novel idea of our research is to demonstrate the role of circSERPINA3 in the regulation of miR-944 gene expression in hepatocellular carcinoma and HCV-infected patients’ plasma. RT-qPCR analysis indicated that circSERPINA3 level was significantly upregulated in HCC and HCV-infected cases in comparison to the related controls. In addition, the gene expression level of circSERPINA3 was significantly increased in HCC cases as opposed to the HCV-infected patients. On the other hand, our results confirmed a distinguishable decrease in miR-944 level of HCC and HCV-infected patients much more than the corresponding controls leading to a significant increase in plasma levels of MDM2; the downstream gene of miR-944 in HCC and HCV -infected patients.

Previous reports approved that MDM2 promotes tumor progression and metastasis in ovarian cancer through ubiquitination of p53 and stimulation of epithelial-to-mesenchymal transition EMT [33]. By suppression of MDM2; microRNA-758-3p inhibits the HCC progression as reported by Jiang et al. (2017) [34]. Hassan et al. (2023), reported that non-coding RNAs are valuable diagnostic markers because they are highly stable and tissue-specific; miR-1290 had high sensitivity and specificity to the HCC cases developed as a progression of HCV viral infection [25].

Accordingly, this study confirmed that the gene expression level of circSERPINA3 in HCV-infected and HCC patients significantly had a negative correlation to the level of microRNA-944 and significantly had a positive correlation to MDM2 protein level, the downstream target of miR-944, in addition to the plasma level of miR-944 showed negative correlation to the oncogenic protein (IGF-1) in HCC patients. Also, this study confirmed that circSERPINA3 upregulation is in correlation with the exaggerated oncogenic IGF-1 pathway in HCC cases because it was negatively correlated to the IGF-1 protein concentration.

Moreover, HCC patients with a significant increase in circSERPINA3 gene expression showed a remarkably lower 1- year survival rate, about 10.75% of HCC patients survived. All these results indicate poor prognosis for patients with high concentration levels of circSERPINA3 and low concentration levels of miR-944.

Thus, our study outcomes are compatible with the findings stated formerly by Liu et al. (2020), that circSERPINA3 could inhibit the action of microRNA-944 by sponging and MDM2 oncogenic effect was activated by circSERPINA3 and overturned by miR-944 [20]. According to Lv et al. (2019), the upregulation of IGF-1R level in Hep3B cells was opposed by microRNA-944 activation and silencing of the same microRNA activated the IGF-1R pathway [16].

According to Yuan et al. (2020), one of the main challenges in the treatment of HCC is metastasis. A small number of HCC patients with advanced metastases could be survived from cancer through surgery and the standard treatment protocols. Epithelial–mesenchyma transition (EMT) plays a vital role in tumor metastasis by losing one of the cellular adhesion proteins like E-cadherin [35].

The immunoblotting investigation was conducted to find out the expression level of E-cadherin, one of the most important epithelial metastasis biomarkers. As shown in our results, the plasma level of E-cad was considerably increased in HCC and HCV-infected cases as opposed to their controls and the level of E-cadherin was significantly higher in HCC cases than in the HCV-infected patients. In addition, E-cad level was remarkably negatively correlated to the gene expression level of microRNA-944 and positively correlated to the gene expression level of circSERPINA in both HCV-infected and HCC patients.

These results demonstrated that miR-944 inhibited the metastasis initiation but the upregulation of circSERPINA3 gene expression reversed the function of miR-944 causing increased expression level of E-cadherin and exaggeration of tumor proliferation and metastasis. Similar to Chen et al. (2021), study results, miR-944 silencing enhances the metastasis through distinctly increased expression of E-cad in cervical cancer [36] besides the increased expression of E-cad in viral hepatitis C is considered a pivotal sign of the development of HCC [17]. E-cadherin increased protein expression level indicates metastasis in hepatic cancer and is associated with HCV-induced EMT, providing a valuable link between the HCV infection and the development of HCC as stated by Yuan et al. (2020) [35]. HCV infection promotes the production of E-cadherin and transforming Growth Factor β-1 (TGF-β1) that activate the hepatocytes' transition from epithelial cells to myofibroblasts during the EMT-inducing liver carcinogenesis. The activation of Twist2, the NS5A HCV proteins can cause EMT transition and metastasis. However, the HCV core viral protein controls the Wnt/-catenin pathway and interacts with the Snail protein to create a complex that enhances the release of E-cadherin from the liver epithelial cells leading to the promotion of HCV infection and inflammation to HCC [37].

In our study, the ROC analysis was constructed to validate the diagnostic ability of the previous biomarkers in the diagnosis of hepatocellular carcinoma.

E-cadherin showed high diagnostic accuracy values in HCV viral infection as the AUC was 0.9167 (P < 0.001). Also, we conducted the ROC analyses to evaluate the diagnostic efficacy of circSERPINA3 and miR-944 in the diagnosis of both hepatitis C and HCC. The results showed high diagnostic accuracy values of the two molecular markers circSERPINA3 and miR-944 in HCC and HCV-infected patients suggesting that circSERPINA3 and miR-944 could be new potential diagnostic biomarkers and therapeutic targets of hepatitis C infection and HCC.

Also, we conducted the ROC analyses to evaluate the diagnostic efficacy of the gene expression levels of circSERPINA3, and miR-944 in the diagnosis of both hepatitis C and HCC. The results showed high diagnostic accuracy values of the two molecular markers circSERPINA3 and miR-944 in HCC and HCV-infected patients suggesting that circSERPINA3 and miR-944 could be new potential diagnostic biomarkers and therapeutic targets of hepatitis C infection and HCC.

Besides, the potential role of molecular biomarkers (miR-944 and circSERPINA3) in the diagnosis of HCC; glypican3 contribution to HCC diagnosis couldn’t be neglected.

Glypican-3 is a member of glypican proteoglycans linked to the cell surface by a glycosylphosphatidylinositol anchor, and acts as a co-receptor that lowers the ligand concentration by enhancing the formation of ligand-receptor complexes [38]. GPC3 has dual roles, a tumor suppressor protein in lung, ovary, and breast organs and an oncogenic protein in tissues with no expression in adulthood as in HCC. Alpha-fetoprotein was considered the main biomarker for HCC diagnosis but its sensitivity and specificity are not enough to provide an accurate diagnosis [39].

A previous study carried out by Motawi et al. (2019), demonstrated that glypican-3 level is elevated in a large percentage of HCC patients as it promotes metastasis and decreases the apoptosis signaling proteins [22] and so, Shih et al. (2020), proposed that glypican-3 has higher diagnostic accuracy and sensitivity than alpha-fetoprotein in the initial diagnosis of HCC and it is an important marker to differentiate between benign liver diseases and the malignant HCC cases [40]. The ELISA results reported that the average concentration of glypican-3 in the HCC group was remarkably increased by 339.4 ± 34.38 pg/ml in comparison to the HCV-infected cases and their controls. Thus, GPC3 could be a diagnostic marker of hepatocarcinogenesis.

Furthermore, we evaluated the diagnostic performance of GPC3 in HCC cases by analysis of the ROC curve, which showed GPC3 had high sensitivity and specificity values (100%). These results were significantly higher than the specificity and sensitivity values of AFP (67.69 and 96.15%) correspondingly in HCC patients that means glypican-3 is an essential diagnostic marker of HCC at the initial stage. Our study results were in line with the Wang & Wei (2020) study, AFP diagnostic accuracy ranges between 41% and 65% with lower specificity than GPC-3 in HCC diagnosis (80%–94%) [41].

Notably, there was a distinctive positive correlation between the plasma GPC3 level in hepatocellular carcinoma patients and the concentration of IGF-1R which means targeting of GPC3/IGF-1 axis could be used to treat HCC.

Activation of the oncogenic IGF-1R pathway by GPC3 leads to the initiation and maintenance of the tumor through inhibition of apoptosis and G1 cell cycle development [42]. Also, Cheng et al. (2022), study reported that the binding of GPC3 to IGF-1R through its N-terminal proline-rich domain, induces the phosphorylation of IGF-1R, extracellular signal-regulated kinase (ERK) and *c*-Myc expression causing cell proliferation and tumor progression in addition to, activation of AKT pathway that leads to protein synthesis and growth [43].

In conclusion, our study revealed that circSERPINA3 gene expression is upregulated in both HCV-infected and HCC patients causing suppression of the antitumor effect of miR-944 and increase of the MDM2 plasma level that aggravates the metastasis, oxidative stress, and cell proliferation in HCC cases. In addition, the downregulation of miR-944 improves the progression of viral hepatitis C cases to hepatocarcinogenesis through increased E-cadherin concentration. GPC3 has greater diagnostic accuracy than AFP in HCC diagnosis besides its pivotal role in the activation of the common HCC oncogenic pathway IGF1/ERK/AKT.

These observations recommend that the newly studied circSERPINA3 and miR-944 molecular biomarkers in HCV-infected and HCC cases could be potential targets for therapeutic opportunities for HCC and HCV-infected patients.

## Ethics approval and patient consent

This research was agreed upon by the local ethical board in Kasr Al-Ainy Hospitals according to the ethical guiding principles of the 1975 Declaration of Helsinki. All participants signed informed consent prior to the donation of blood according to the ethical standard laid down in the Helsinki Declaration and current national laws, as well as approved by the Research Ethics Committee at Faculty of Pharmacy, MSA University, Cairo, Egypt. (Approval number: Bp1/EC1/2020MSC).

## Funding

The authors declare that they had received no funding for the research reported.

## Availability of data and materials

All data generated or analyzed during this study are included in this published article.

## CRediT authorship contribution statement

**Nora M. Aborehab:** Conceptualization, Data curation, Formal analysis, Material preparation. **Mohamed A. Kandeil:** Conceptualization, Material preparation. **Dina Sabry:** Conceptualization, Material preparation. **Radwa Rabie:** Data curation, Formal analysis, Writing – original draft. **Ibrahim T. Ibrahim:** Conceptualization, Data curation, Formal analysis, Material preparation, All authors read and approved the final manuscript, All authors contributed to the study's conception and design.

## Declaration of competing interest

The authors declare that they have no known competing financial interests or personal relationships that could have appeared to influence the work reported in this paper.

## Abbreviations

****Abbreviation Explanation****
AFP: Alpha feto-protein
Akt: Protein kinase B
ALP: Alkaline phosphatase
ALT: Alanine aminotransferase
AST: Aspartate amino transferase
BSA: bovine serum albumin
circ LARP4: Circular RNA La-related RNA-binding protein 4
circMTO-1: Circular mitochondrial translation optimization 1 homolog
CircRNA: Circular RNA
CT scan: Computed Tomography Scan
E- Cad: E-Cadherin
ELISA: enzyme-linked immunosorbent assay
EMT: epithelial-to-mesenchymal transition
ERK: Extracellular signal-regulated kinase
GPC3: Glypican-3
HCC: Hepatucellular carcinoma
HCV: Hepatitis C virus
IGF-1R: Insulin growth factor-1 receptor
MDM2: Mouse double minute 2 homolog
miRNA: MicroRNA
MNCs: Mononuclear cells
MRI scan: Magnetic resonance imaging
PI3K: Phosphatidylinositol-3 kinase
RIPA: Radioimmunoprecipitation assay
RT- PCR: Reverse transcription polymerase chain reaction
SDS-PAGE: Sodium dodecyl-sulfate polyacrylamide gel electrophoresis
SERPINA3: Serine proteinase inhibitor A3
T Bil: Total bilirubin
WBCs: White blood cells
