# Supplementary material for: Circular SERPINA3 and its target microRNA-944 as potential biomarkers in hepatitis C virus-induced hepatocellular carcinoma in Egyptian population
Source: Noncoding RNA Res. 2023 May 23;8(3):401–12. doi: 10.1016/j.ncrna.2023.05.005 (PMC10247953; doi:10.1016/j.ncrna.2023.05.005)
Supplement: Multimedia component 1 [file mmc1.docx]

**Supplementary material**

**Table S1. Comparison of the serum gene expression levels of circSERPINA3 and miR-944 among the studied groups**

| **Marker** | **Control** | **HCV** | **HCV + HCC** |
| --- | --- | --- | --- |
| **CircSERPINA3** | 1.756 ± 0.2258 | 3.956 ± 0.09579*** ^a^ | 6.212 ± 0.2653*** ^ab^ |
| **miR-944** | 4.135 ± 0.1343 | 1.917 ± 0.1436*** ^a^ | 1.681 ± 0.09667*** ^a^ |

| **Marker** | **Control** | **HCV** | **HCV + HCC** |
| --- | --- | --- | --- |
| **MDM2** | 0.6409 ± 0.04351 | 0.9948 ± 0.1229 | 4.339 ± 0.2372*** ^ab^ |
| **E-cadherin** | 27.31 ± 1.317 | 55.10 ± 3.626*** ^a^ | 61.50 ± 5.793*** ^ab^ |
| **Glypican-3** | 102.2 ± 3.935 | 114.5 ± 6.781 | 339.4 ± 34.38***  ^ab^ |

**Supplementary Table S1. The RT-PCR expression levels of the serum circSERPINA-3 and miR-944 in the studied groups.** Gene expression levels are expressed as mean ± SEM. The data were evaluated using ANOVA and Tukey Kramer’s multiple comparison tests for differentiation between the three studied groups. a statistically significant from healthy controls. b statistically significant from HCV. ***Indicates significance at p < 0.001.

**Table S2. Comparison of the serum MDM2, E-cadherin and GPC3 expression levels among the studied groups**

**Supplementary Table S2. The protein expression levels of the plasma MDM2, E-cadherin and GPC3 measured by western blotting and ELISA in the studied groups.** The protein expression levels are expressed as mean ± SEM. The clinical data were analyzed by ANOVA and Tukey Kramer’s multiple comparison test for differentiation between the three studied groups. ^a^ statistically significant from healthy controls. ^b^ statistically significant from HCV. ***Indicates significance at p < 0.001.

**Supplementary Figure S1:** Western blot for MDM2 protein expression.

| **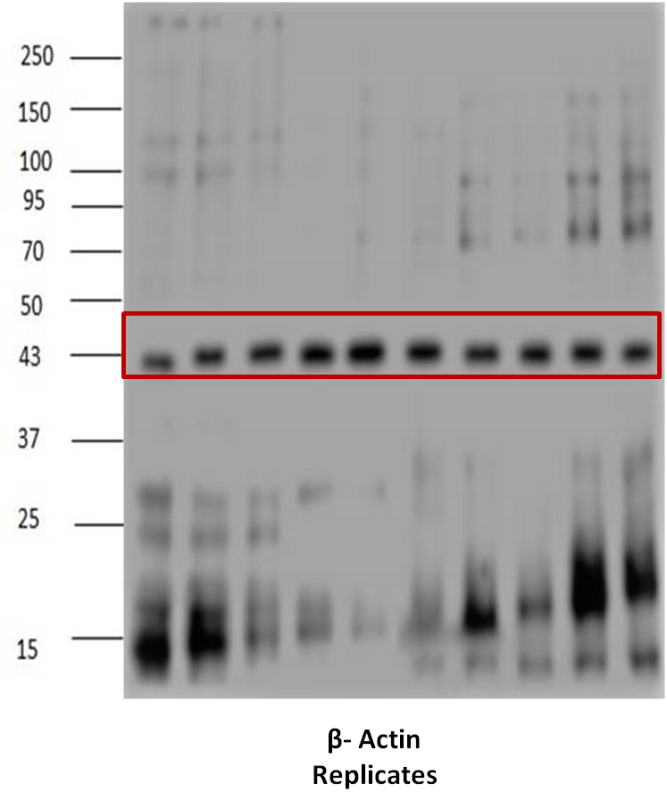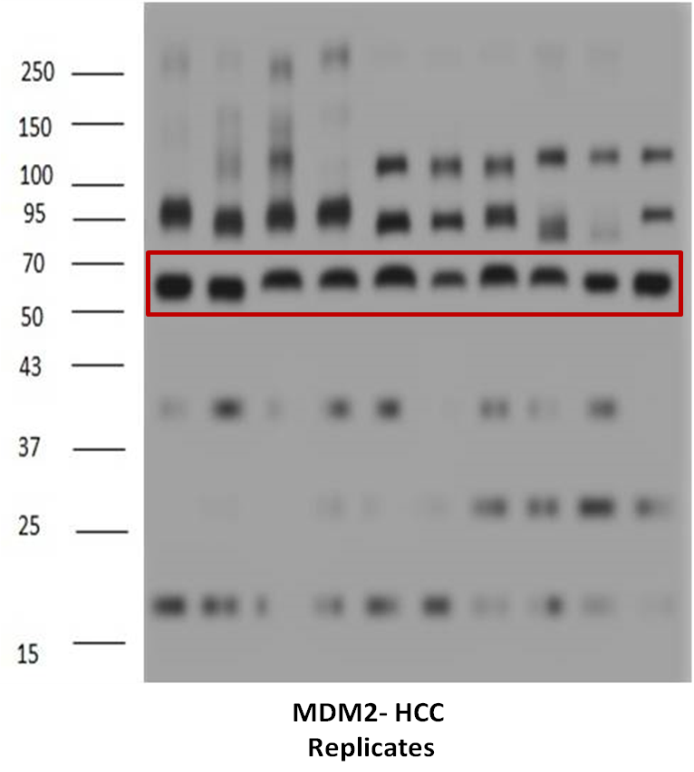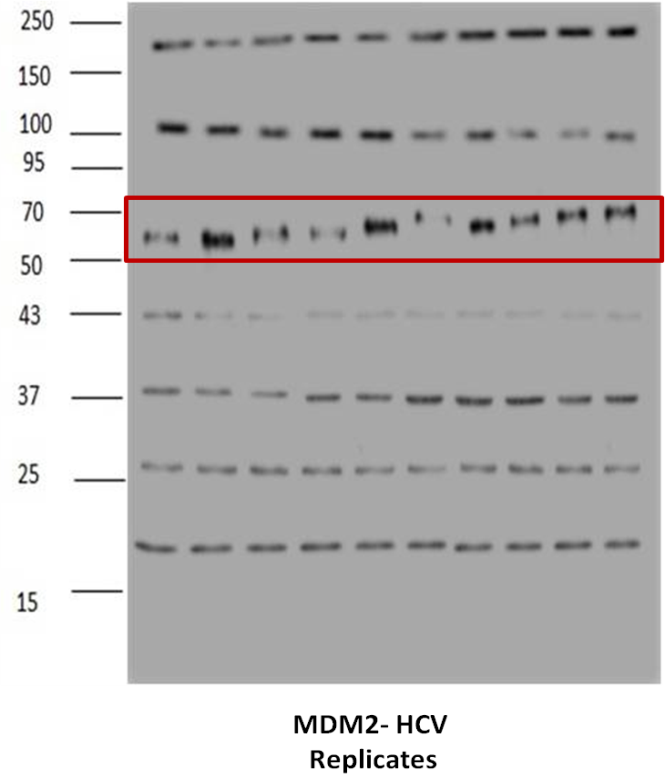**    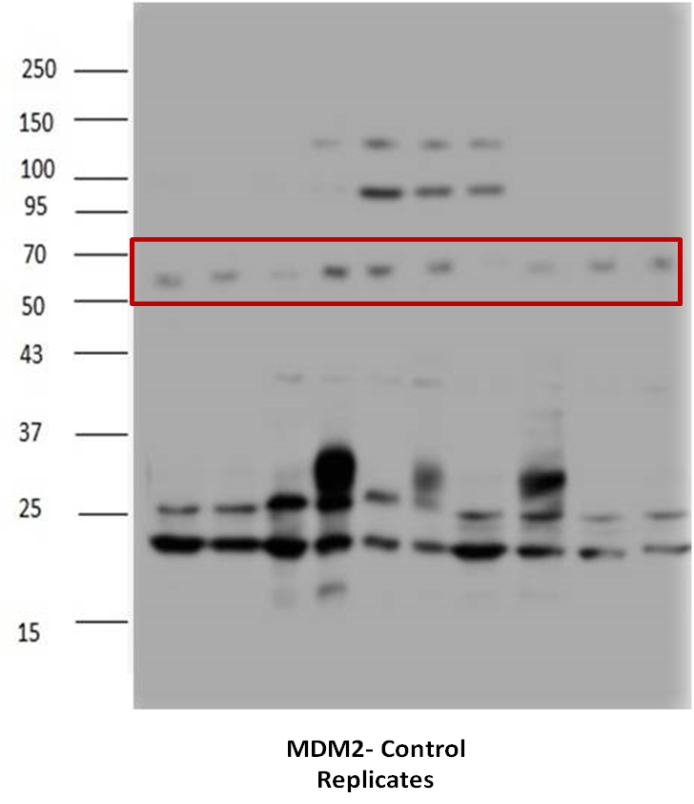 |
| --- |

**Supplementary Fig.S1.** Protein expression levels of MDM2 as analyzed by Western blotting at molecular weights (60 kDa) for MDM2 and (43 kDa) for β-actin. HCV, hepatits C virus; HCC, hepatocellular carcinoma; MDM2, mouse double minute 2 homolog.

**Supplementary Figure S2:** Western blot for E-cadherin protein expression.

| **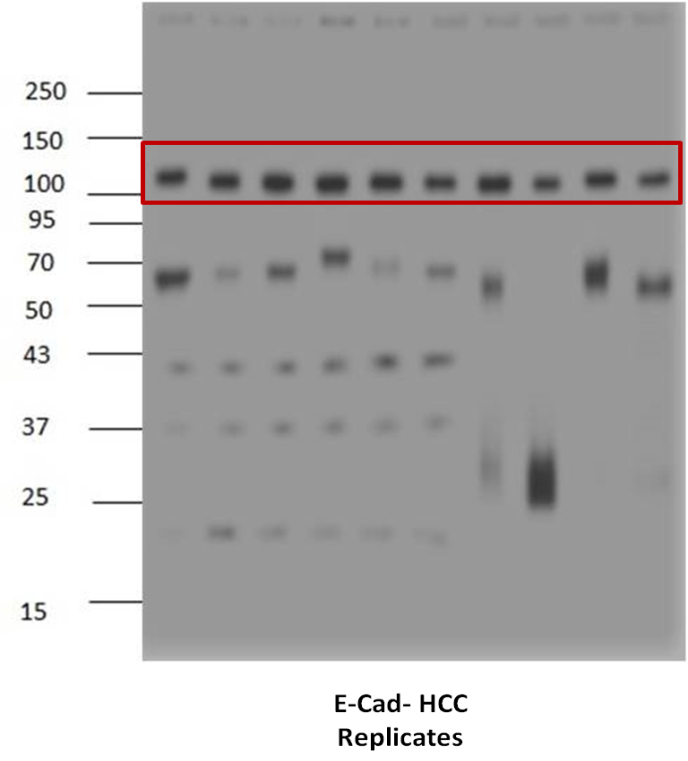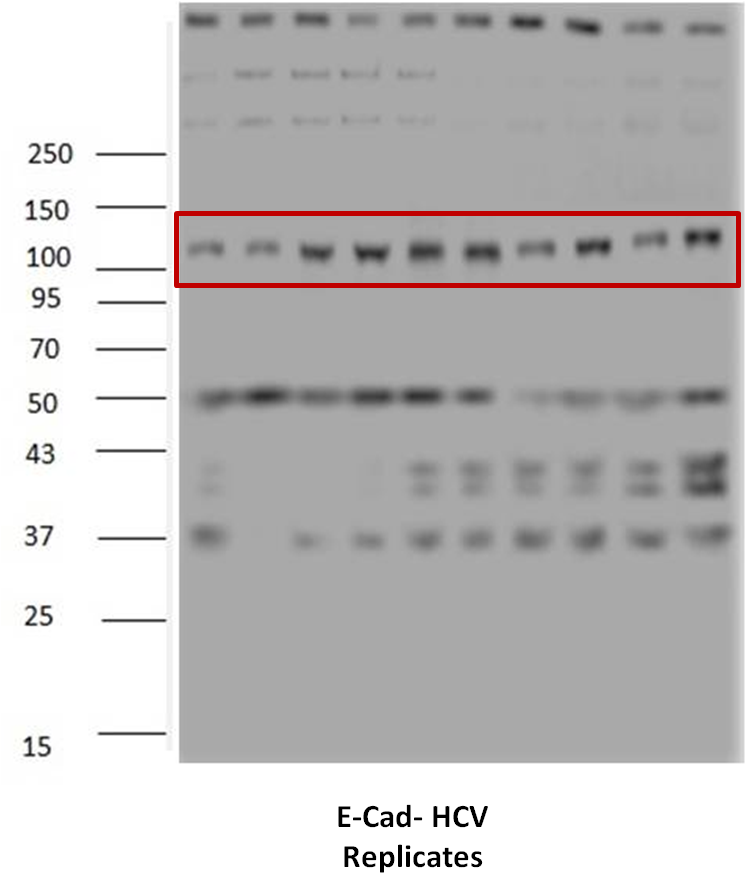**  **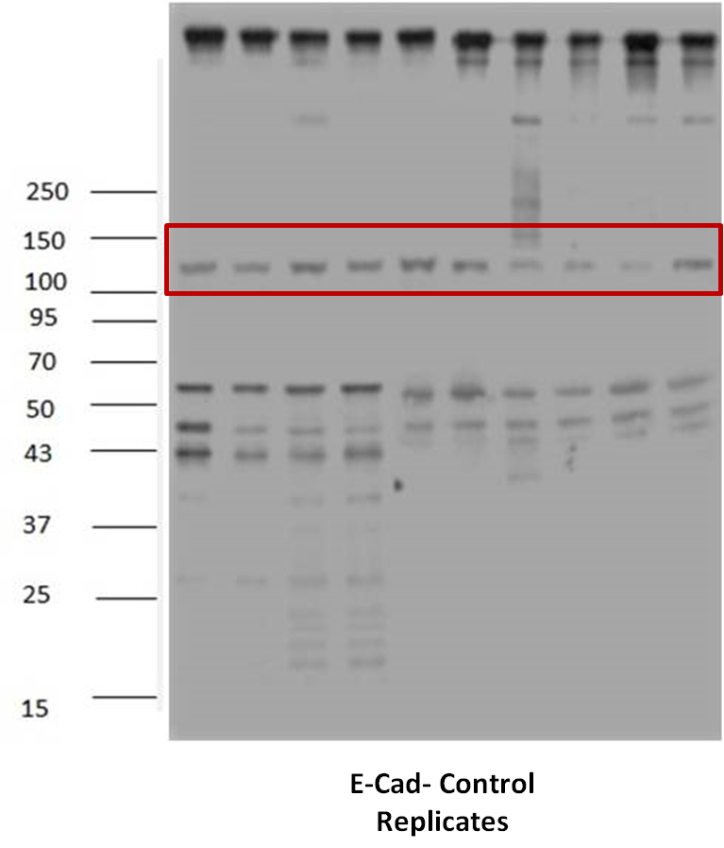**  **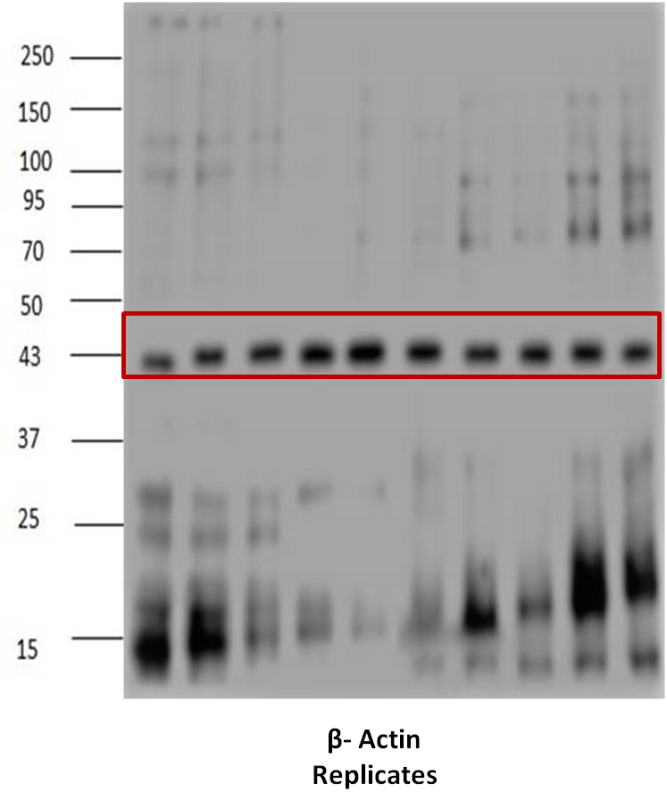** |
| --- |

**Supplementary Fig.S2.** Protein expression levels of E-cadherin as analyzed by Western blotting at molecular at molecular weights (135 kDa) for E-cadherin and (43 kDa) for β-actin. HCV, hepatits C virus; HCC, hepatocellular carcinoma;E-Cad, E-cadherin.
